# High tibial osteotomy for acute correction and subsequent gradual tensioning of the posterolateral knee ligament complex in treating genu varum combined with a lateral thrust using the Ilizarov technique in adults: surgical technique and early results

**DOI:** 10.1186/s13018-023-03900-8

**Published:** 2023-06-10

**Authors:** Mohamed Abdelaal Hussein, Ahmed A. Khalifa, Gamal Ahmed Hosny

**Affiliations:** 1 Orthopaedic Department, National Institute of Neuromotor System, Cairo, Egypt; 2 Orthopaedic Department, Qena Faculty of Medicine and University Hospital, South Valley University, Kilo 6 Qena-Safaga Highway, Qena, 83523 Egypt; 3 Orthopaedic Department, Benha University, Benha, Egypt

**Keywords:** High tibial osteotomy, Lateral thrust, Ilizarov, Posterolateral corner

## Abstract

**Objective:**

To report the early results of using the Ilizarov technique in performing medial wedge opening high tibial osteotomy (MWOHTO) combined with gradual tensioning of the posterolateral corner in adult patients presenting with genu varum (GV) and lateral thrust.

**Methods:**

A prospective case series study included 12 adult patients with a mean age of 25.2 ± 8.1 years who presented with GV deformity associated with lateral thrust. They were evaluated clinically using the “hospital for special surgery” (HSS) knee scoring system. Radiological evaluation was performed using long film from hip to knee to ankle (HKA) radiographs; the overall mechanical alignment was measured as the HKA angle, the upper tibial deformity was measured as the medial proximal tibial angle (MPTA), and the joint line convergence angle (JLCA) was measured. Surgical technique included using Ilizarov for MWOHTO below the level of the tibial tubercle, acute correction of the GV deformity, fibular osteotomy, and gradual distalization of the proximal fibula.

**Results:**

After a mean follow-up of 26.3 ± 6.4 months, all osteotomies were united. All patients achieved fibular osteotomy site bony union except two with a fibrous union. The HSS score showed improvement from a mean preoperative score of 88.7 ± 7.6 to a postoperative 97.3 ± 3.9 (*P* < 0.05). The overall mechanical lower limb alignment improved significantly from a mean preoperative HKA of 164.5 ± 3.2 to a postoperative 178.9 ± 1.6 (*P* < 0.05). The MPTA improved significantly from 74.6 ± 4.1 to 88.9 ± 2.3, as well as the JLCA from 12.17 ± 1.9 to 2.3 ± 1.7 (*P* < 0.05). Grade 1 pin tract infection was developed in four patients and was treated conservatively. In two patients, mild pain over the fibular osteotomy site was relieved over time. The lateral thrust reoccurred at the last follow-up evaluation in the two poliomyelitis patients.

**Conclusion:**

MWOHTO, concomitant with tensioning the knee lateral soft tissue structure at the same setting through applying an Ilizarov apparatus, showed promising functional and radiological outcomes.

## Introduction

High tibial osteotomy (HTO) is a knee-joint-preserving procedure that is indicated to correct either varus or valgus deformity of the knee and shows acceptable long-term outcomes [1–4]. In patients with genu varum (GV), either if the knee is arthritic or not, various varieties of HTO could be performed, including medial wedge opening (MWO), lateral wedge closure (LWC), or dome osteotomy, aiming at shifting the mechanical axis from medial to neutral or slightly lateral to offload the medial compartment, correct deformity, and improve the lower limb kinematics [2, 5]. Furthermore, MWOHTO could be performed below or above the tibial tuberosity (TT), and various fixation devices were used to stabilize the osteotomy site, such as plates and screws, monolateral external fixator, and Ilizarov apparatus [5–8].

Knee GV deformity could be associated with a lateral thrust, defined as a dynamic worsening or an abrupt onset of the knee varus deformity upon weight bearing and during walking. This indicates a dynamic posterolateral knee instability with increased loading of the medial knee compartment [9, 10]. Lateral thrust was attributed to various factors, including ligament mal-balance or laxity and quadriceps weakness; however, the exact reason for its occurrence is still poorly identified [9, 11].

It is well documented that the knee lateral soft tissue structures, mainly the posterolateral corner (PLC), are under over-tension and progressive laxity in GV deformity either in adults or in the pediatric population, with subsequent joint line convergence and knee instability [12, 13]. Some authors suggested that lateral soft tissue laxity will improve over time after HTO; however, persistent lateral thrust could negatively affect the knee in the form of cartilage damage, worsening of pain, and bone marrow lesions; thus, its correction is mandatory to preserve the knee joint integrity, especially in non-arthritic knees [3, 14–16].

So correcting GV and lateral thrust should be aimed for during surgery. In the current case series, we described performing MWOHTO below the level of the TT and fixation using the Ilizarov apparatus in adult patients presenting with GV and an evident lateral thrust. In the same setting, Ilizarov was used to re-tension the knee PLC structures through fibular osteotomy and distalization of the proximal part of the fibula. We hypothesized that this technique would achieve deformity correction and knee stability by gradually tensioning the knee lateral soft tissue structures at the same procedure without further consequences or the need for secondary procedures.

## Patients and methods

This prospective case series study included 12 adult patients (older than 16 years) presented with genu varum deformity associated with a lateral thrust between January 2018 and December 2021. We excluded patients with post-traumatic deformity and advanced arthritic changes of the medial knee compartment. The mean age of the patients was 25.2 ± 8.1 years, 10 were males, two were females, and two had a post-poliomyelitis deformity.

Clinical knee evaluation was performed using the “Hospital for Special Surgery” (HSS) knee scoring system pre- and postoperatively [17]. The lateral thrust was assessed clinically by one of the authors, where patients were observed walking away and then back to the assessor for 10 m, and the lateral thrust was classified as either definitely present, possibly present, or definitely absent, as described in previous studies [18, 19], where all patients in the current series had definitive lateral thrust. Further clinical assessment of the knee was performed to detect possible anteroposterior instability (indicating ACL or PCL deficiency) [20].

Radiological evaluation was performed using long film, hip to knee to ankle (HKA) radiographs, and the following parameters were assessed both pre- and postoperatively: the overall mechanical alignment was measured as the HKA angle, the upper tibial deformity was measured as the medial proximal tibial angle (MPTA), and the joint line convergence angle (JLCA) was measured [21, 22].

### The operative technique (Figs. 1 and 2)

**Fig. 1:**
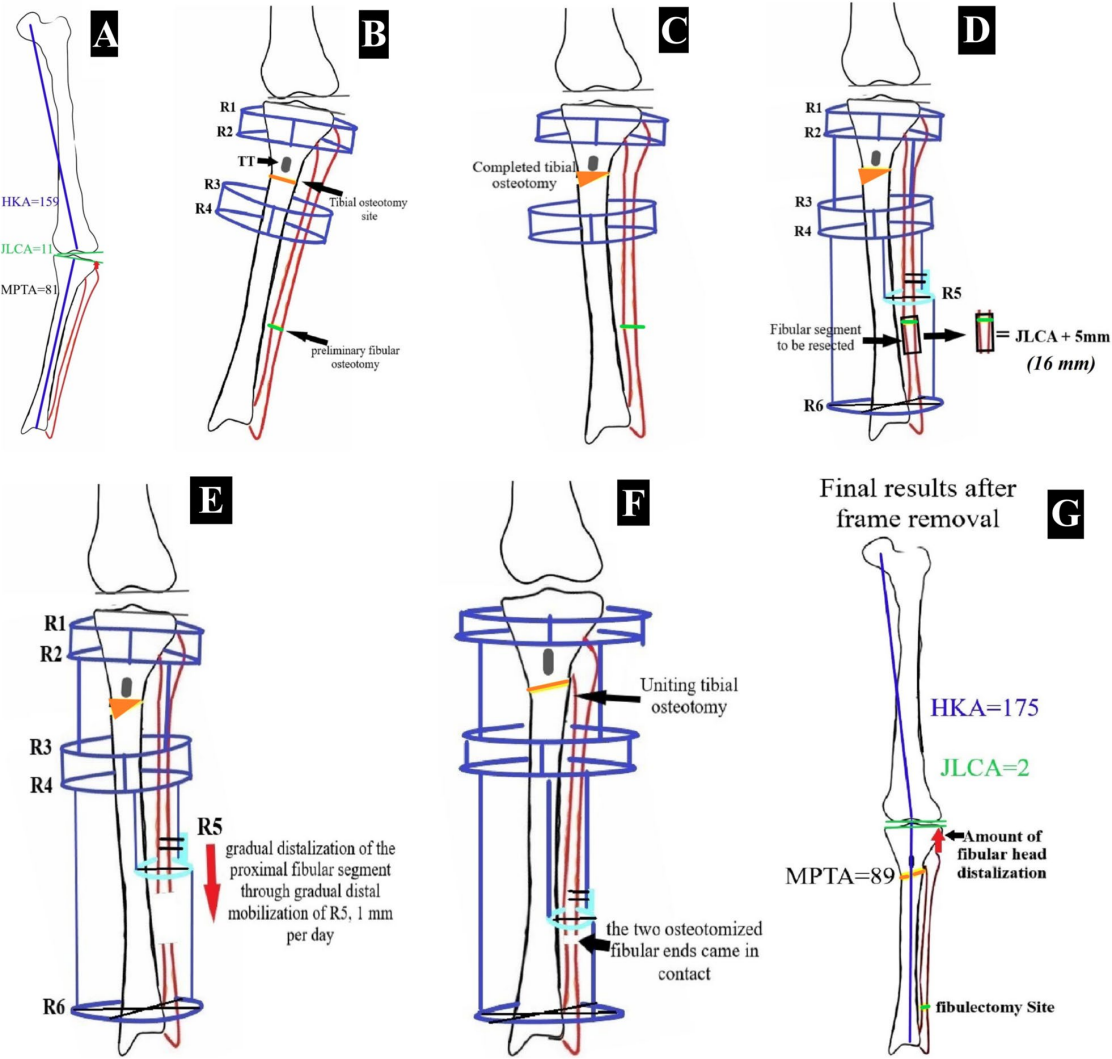
Schematic diagram showing the operative technique details. **A** Preoperative planning on a long film (hip to knee to ankle). **B** Locating the osteotomy below the tibial tuberosity after application of two rings above (R1 and R2) and two below (R3 and R4) and a preliminary transverse fibular osteotomy. **C** After completing the tibial osteotomy, full correction is achieved, and the frames are connected. **D** Application of a ring above the fibular osteotomy site (R5) and a ring at the ankle level (R6), which are connected to the rest of the frame, and a block is resected from the fibula. **E** Starting gradual distalization of the proximal fibular segment through distal mobilization of R5 (1 mm per day, starting 7 days postoperatively). **F** The tibial osteotomy is uniting, and the site of the fibular osteotomy is nearly closed. **G** Final images after obtaining full union at the tibial and fibular osteotomies sites, and removal of the hardware, showing the deformity correction and the among of fibular head distalization

**Fig. 2:**
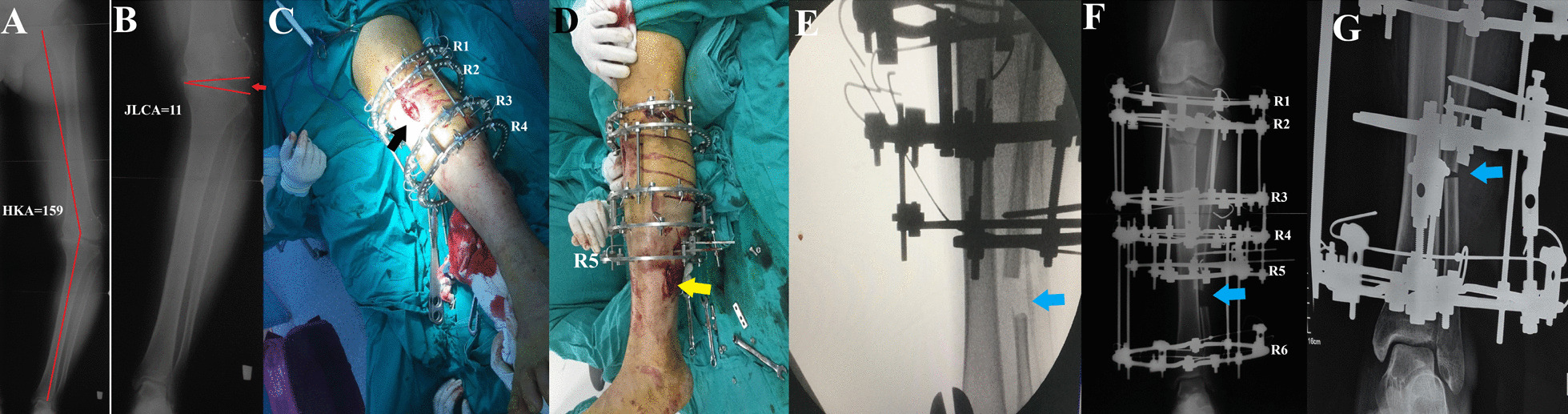
Male patient, 36 years old, presented with left knee pain and genu varum deformity. **A** preoperative long HKA radiograph showing varus deformity of 159°. **B** Joint line convergence angle of 11°. Intraoperative, **C** After applying the Ilizarov and performing the medial wedge opening high tibial osteotomy (black arrow). **D** After performing the fibular osteotomy (yellow arrow showing the incision site). **E** Intraoperative fluoroscopic image showing the site of the fibular osteotomy (blue arrow). **F** and **G** Follow-up radiographs showing closing of the fibular osteotomy gap (blue arrow) after gradual distalization of the proximal fibula

Preoperative planning was performed for all patients using a long AP film, including the hip, knee, and ankle; the overall mechanical limb alignment was measured as the HKA angle, which is the medial angle between the mechanical axis of the femur and the mechanical axis of the tibia. Furthermore, the joint line convergence angle (JLCA), the angle between the distal femoral and proximal tibial joint lines, was measured. The site of the osteotomy and the reconstruction of the Illizarov rings (details of which are described later) needed during surgery were all planned preoperatively.

The senior author, an experienced pediatric and deformity correction surgeon, performed all the operative procedures. Surgeries were performed under spinal anesthesia, while the patient was supine on a radiolucent table. A thigh tourniquet was applied. Draping was performed in a way not to block the image intensifier from accessing the ipsilateral hip joint for later assessment of the mechanical alignment using the cable technique. Detailed surgical steps were as follows:

*First*, Tibial rings fixation:

- ring 1 (R1), a 5/8 ring at the tibial metaphyseal area just below the joint line,
- ring 2 (R2), full ring below the first one and above the TT,
- ring 3 (R3), full ring below the TT,
- ring 4 (R4), full ring below R3.

*Second*, a preliminary simple transverse fibular osteotomy was performed at the junction between the middle and distal thirds of the fibula to facilitate the correction through the tibial osteotomy. Then, the proximal tibial osteotomy was performed through a 3-cm midline skin incision, 1–2 cm distal to the TT, using a drill and osteotome technique. Complete deformity correction was achieved by applying a valgus force to the distal fragment until full correction to a neutral mechanical alignment. This was assessed under the image intensifier using the cable method, which was stretched from the femoral head center to the ankle joint center and should pass through the center of the knee (or to an alignment equal to the contralateral limb if normal). The rings were then connected to secure the osteotomy site.

*Third*, ring 5 (R5) is a 5/8 ring (smaller in size than R1) placed laterally just above the fibular osteotomy level and below the level of R4, fixed to the fibula using a 1.8-mm wire and two 3-mm half pins.

*Fourth,* ring 6 (R6) is a full ring placed just above the level of the ankle joint, fixed by three wires transfixing the tibia and fibula, which was connected to R4 using rods.

*Fifth*, the fibular osteotomy was readdressed, and a block was removed, the length of which is determined according to the preoperatively measured JLCA plus 5 mm (bone block segment length = JLCA + 5 mm), where the JLCA represents the amount of slackness of the PLC and the added 5 mm to provide over tensioning of the soft tissues.

*Sixth*, the R5 was connected to the ring above (R4) and below (R6) by rods and plates.

*Last*, distalization of the proximal fibular segment through gradual distal mobilization of R5 started 1 week postoperatively, and the distraction amount was 1 mm per day divided into four increments (0.25 mm every 6 h) until the two osteotomized ends came in contact.

### Postoperative protocol

In the hospital and under the supervision of a physiotherapist, patients were allowed to full weight bearing using two crutches from the first day postoperatively. A full range of motion was allowed for the knee and ankle, accompanied by strengthening exercises of the quadriceps muscles and ankle dorsi- and plantar flexors. Patients usually are discharged on the second postoperative day, before which they are educated regarding the daily care of the frame and the wires and were instructed regarding early signs of pin-related infection.

Follow-up visits were scheduled at 2 weeks for sutures removal and preliminary radiographic check of the frame position and alignment; if any re-adjustment was needed, it was performed at this time. Then, the visits were every 2 weeks till the osteotomy union was achieved, then every 3 months during the first postoperative year and then annually. The frame was only removed after the complete HTO site union, which was assessed in the serial follow-up radiographs and clinically over the osteotomy site.

Functional and radiological outcomes were reported at the last follow-up. The time the frame was applied was reported, and the time till upper tibial osteotomy union. Any complications (including the fibular osteotomy site and the proximal fibula) were reported and classified as minor or major according to Paley's classification [23].

Statistical analysis: Statistical analyses were performed using SPSS (SPSS for Windows Release 15.0; SPSS Inc., Chicago, IL, USA). Data were presented as averages and standard deviations. Mann–Whitney U test was used to compare outcomes pre- and postoperative, and statistically significant results were considered if the *P* value was < 0.05.

## Results

**Fig. 3:**
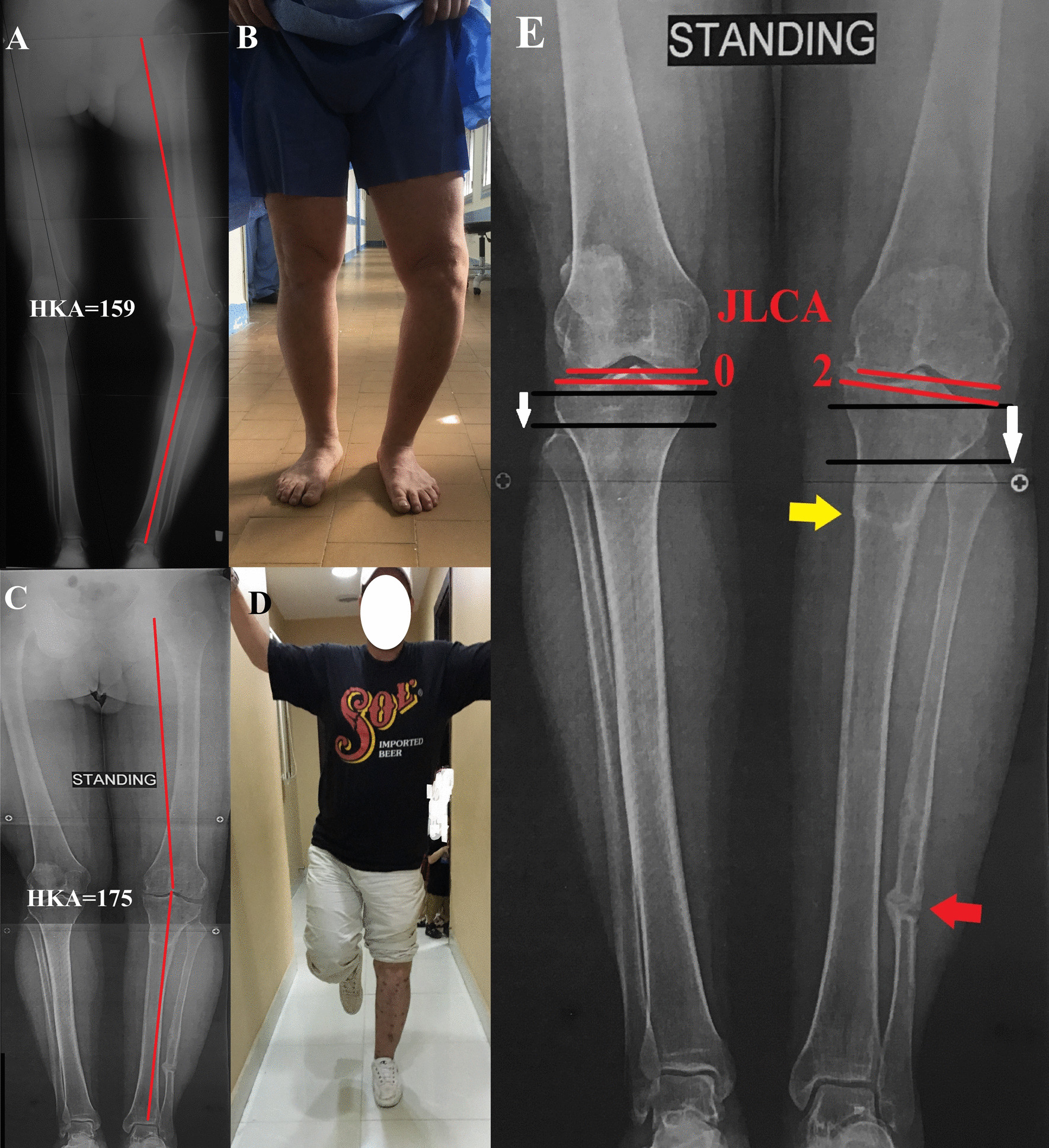
The same patient in Fig. 1. **A** preoperative radiograph, **B** clinical image while the patient is standing showing the amount of genu varum deformity. At the final follow-up at 18 months, **C** long film radiograph showing correction of the deformity to an HKA of 175°, **D** clinical image while the patient is standing showing deformity correction and indicating knee stability. **E** comparing the operated knee (left) with the normal side showing near normal JLCA (red lines), healing of the tibial osteotomy (yellow arrow), healing of the fibular osteotomy (red arrow), and the amount of fibular head distalization compared to the normal side (white arrows)

All patients were available for evaluation. After a mean last follow-up of 26.3 ± 6.4 months, all patients had HTO site union, and the frame was removed after a mean of 15.3 ± 2.9 weeks. All patients achieved fibular osteotomy site bony union except two with a fibrous union. The clinical assessment according to the HSS score showed improvement from a mean preoperative score of 88.7 ± 7.6 to a postoperative 97.3 ± 3.9 (*P* < 0.05); the score was excellent in eight patients, good in two, and fair in two (diagnosed preoperatively as having poliomyelitis). The clinical evaluation of the lateral thrust was definitely absent in 10 (83.3%) patients. The overall mechanical lower limb alignment improved significantly from a mean preoperative HKA of 164.5 ± 3.2 to a postoperative 178.9° ± 1.6° (*P* < 0.05). The MPTA improved significantly from 74.6° ± 4.1° to 88.9° ± 2.3°, as well as the JLCA from 12.17° ± 1.9° to 2.3° ± 1.7° (*P* < 0.05) (Fig. 3).

Regarding complications, we did not encounter any major complications, DVT, neurovascular complications, or correction failure. However, grade 1 pin tract infection was developed in four patients and was treated conservatively. In two patients, mild pain over the fibular osteotomy site; however, the pain subsided over time. In the two (16.7%) post-poliomyelitis cases, the lateral thrust reoccurred at the last follow-up evaluation and was graded as possibly present.

## Discussion

Knee ligamentous laxity or insufficiency in concomitant with GV used to be a contraindication for HTO; this concept was changed over time as biomechanical studies showed improved knee stability after HTO [24]. Furthermore, in knees having chronic deficient PLC, ACL, or both, what is called double or triple varus knees, respectively [25, 26]. HTO can improve load imbalance with the improvement in axial alignment, thus reducing the varus thrust, improving pain, and postponing the progression or development of knee osteoarthritis, especially when dealing with active young patients [3, 14–16]. Besides, if ligamentous reconstruction was decided, it should be proceeded by HTO to avoid reconstruction early failure [25].

To our knowledge, the technique described in the current series was not previously discussed in the literature. We achieved acceptable functional and radiological outcomes with minimal complications incidence after using the Ilizarov technique for correcting GV deformity and restoring the overall mechanical alignment acutely. At the same procedure, we improved the lateral knee thrust by restoring the knee lateral soft tissues (PLC) tension without needing later reconstruction through the gradual distalization of the proximal fibula using the same device.

PLC is crucial for knee stability in the frontal plane, especially in preventing excessive tibial external rotation and excessive lateral tibiofemoral opening. Furthermore, its laxity is attributed to the development of lateral thrust [27, 28]. Even though some reports indicated that an isolated HTO could be beneficial for managing acute or chronic knee instability and laxity of the ACL and/or PLC [29, 30], the importance of reconstructing the PLC during HTO for better functional outcomes has been alluded to by some authors [31, 32].

In a study by Helito et al., the authors performed an MWOHTO combined with PLC reconstruction in five patients with chronic PLC injury and GV; they reported a union of the osteotomy in all patients. Although four showed minimal residual instability at the last follow-up, their functional outcomes were satisfactory; the authors stated that this technique benefits active young patients requiring high function [32].

In the current series, we believe that the patients had an intact PLC structure; however, these structures are slackened, lax, and not functioning properly, unlike cases that present with concomitant GV with traumatic PLC injury. So in the current cases, we aimed at restoring the PLC tension through gradual distalization of the proximal fibula to restore the lateral soft tissue tension and avoiding the reported complications and technical difficulty encountered with surgical PLC reconstruction [33, 34].

The current technique provided fewer complications related to the PLC compared to surgical reconstruction. A systematic review evaluated 1747 patients from 60 studies to estimate the incidence of complications after surgical reconstruction of the PLC; the authors reported that the intraoperative complications were low, reaching 2.8%; however, the postoperative complications were high reaching up to 51% in some studies including arthrofibrosis, infection, common peroneal nerve palsy, and reconstruction failure [33]. Furthermore, the gradual tensioning of the PLC provided by Ilizarov avoids undue tension of the common peroneal nerve or its branches, which was suggested to be at risk of injury while drilling the fibular head (during surgical PLC reconstruction) in up to 57% [35].

In the current study, we performed an MWOHTO rather than an LWCHTO. According to a study by Deie et al. [7] comparing various kinematic and functional outcomes between MWO- and LWC-HTO, the authors found that MWOHTO is better in improving the lateral thrust and knee varus moment. Furthermore, they found that in cases where LWCHTO was performed, the lateral thrust reoccurred, the authors explained the difference between the two techniques in maintaining the lateral thrust improvement according to the fact that by opening the medial tibia during MWOHTO this decreases the varus–valgus instability compared to LWCHTO [7].

Good outcomes after using the Ilizarov apparatus for correcting GV deformity have been reported in the literature [8, 36, 37]; furthermore, it carries some advantages. It provides better stability, allowing patients of early full weight bearing to fine-tune and adjust the correction and minimal soft tissue distribution, primarily if the osteotomies are performed percutaneously. As we described, the current technique has the added advantage of addressing the PLC laxity using the same device without requiring direct surgical intervention to the lateral soft tissue complex. However, it also carries some risks; the learning curve was needed for the surgeon to apply the device correctly, the possibility of pin-tract infection, which could affect future total knee arthroplasty if needed, and the bulky device, which could be uncomfortable for the patient.

Although HTO performed above the level of the TT, it has a good potential of union; however, it carries the disadvantages of patella Alta or Baja according to the performed osteotomy type (MWO vs. LWC), potential fixation failure in the narrow metaphyseal area and intraarticular fractures, and could sophisticate the future total knee arthroplasty [1, 4, 16]. In the current study, all HTOs were performed below the TT, and we achieved optimum correction and HTO union in all cases; it carries the advantage of plenty of bone stock for fixation and healing, no disruption of the patellofemoral complex, easier osteotomy execution, and the functional outcomes that had been reported to be satisfying [6, 36–38].

An extra step was added in the current study; the fibular osteotomy had no significant consequences on the overall functional or radiological outcomes. In a study by Deie et al. in the cases where LWCHTO was performed, the authors reported performing an ipsilateral fibular osteotomy with resection of 1.5–2 cm from the mid-fibula with no complications related to the fibular osteotomy [7]. In a cadaveric study by Tanifuji et al. [39] evaluating the effect of adding a fibular osteotomy accompanying the MWOHTO, the authors showed that the medial joint space was wider in MWOHTO with a fibular osteotomy compared to without.

The current study has some limitations: First is the small number of included patients, which underpowers the current results. Second, a detailed evaluation of the knee kinematics and gait analysis was not performed due to a lack of equipment. Third, we should have included a comparative group to indicate the usefulness and superiority of the technique we presented in the current study. Fourth, we included two patients with deformity post-poliomyelitis who could not be good candidates for such a procedure due to quadriceps weakness, issues with knee kinematics, improper ligament tensioning, and poor muscle balance. Last, we did not perform an MRI evaluation of the knee to determine the status of other knee ligamentous structures.

## Conclusion

Medial wedge opening high tibial osteotomy performed below the level of the tibial tuberosity to acutely correct varus deformity and gradual tensioning of the knee lateral soft-tissue complex with the application of the Ilizarov apparatus showed promising clinical and radiological outcomes. The surgical technique is challenging and must be performed by an experienced orthopedic surgeon. A larger sample study is mandatory to test its feasibility and reproducibility and confirm the results obtained in our case series.

## Data Availability

All the data related to the study are mentioned within the manuscript; however, the raw data are available with the corresponding author and will be provided up on a written request.

## Abbreviations

HTO: High tibial osteotomy
GV: Genu varum
MWO: Medial wedge opening
LWC: Lateral wedge closure
PLC: Posterolateral corner
HSS: Hospital for Special Surgery
HKA: Hip to knee to ankle
MPTA: Medial proximal tibial angle
JLCA: Joint line convergence angle
TT: Tibial tuberosity
